# Should knee extension strength testing be implemented as a screening test for identifying probable and confirmed sarcopenia in older T2DM patients?

**DOI:** 10.1186/s11556-021-00280-y

**Published:** 2022-01-27

**Authors:** Ofer kis, Assaf Buch, Roy Eldor, Amir Rubin, Ayelet Dunsky, Naftali Stern, Daniel S. Moran

**Affiliations:** 1 The Faculty of Health Sciences, Ariel University, Ariel, Israel; 2 Institute of Endocrinology, Metabolism and Hypertension, Tel Aviv Sourasky Medical Center, 6 Weizmann St, 64239 Tel-Aviv, Israel; 3 The Sagol Center for Epigenetics of Metabolism and Aging, Tel Aviv Sourasky Medical Center, Tel-Aviv, Israel; 4 School of Health Sciences, Ashkelon Academic College, Ashkelon, Israel; 5 The Academic College at Wingate, Wingate Institute, Netanya, Israel; 6 The Sackler Faculty of Medicine Tel-Aviv University, Tel-Aviv, Israel

**Keywords:** Appendicular skeletal mass index, Handgrip strength, Older adults, Knee extension strength, Sarcopenia, Type 2 diabetes

## Abstract

**Background:**

The accelerated loss of muscle strength and mass observed in older type 2 diabetes mellitus (T2DM) patients due to the combined effects of diabetes and obesity, greatly increases their risk for sarcopenia. Early detection and treatment of probable and confirmed sarcopenia is paramount to delay mobility disability. Using low handgrip strength cut-off points for the initial identification of sarcopenia according to the new European Working Group on Sarcopenia in Older People (EWGSOP2) guidelines may mask the presence of sarcopenia. Relative knee extension strength cut-off points using a simple hand-held dynamometer can assist clinicians in the diagnosis of probable and confirmed sarcopenia by possibly reducing false negative results.

**Methods:**

A cohort of one hundred T2DM older patients (60% women) (mean age 74.5 years) mostly obese community dwelling older adults were evaluated for body composition by Bioelectrical impedance analysis (BIA), yielding appendicular skeletal mass index (ASMI) results. Patients underwent handgrip strength (HGS) and knee extension strength (KES) tests as well as functional ability tests. Prevalence of probable and confirmed sarcopenia using HGS and KES cut-off points were calculated. Pearson correlations were performed to evaluate the relationship between ASMI and limbs strength. A regression analysis was conducted to examine which variables best predict ASMI values. A multivariate analysis of covariance was performed to assess the effect of independent variables on KES and HGS.

**Results:**

Using cutoff points for low KES identified 24 patients with probable sarcopenia and two with confirmed sarcopenia. Conversely, using the EWGSOP2 cut off points for low HGS, identified only one patient with probable sarcopenia and none of the patients with confirmed sarcopenia.

**Conclusion:**

KES cut-off points using a simple hand-held dynamometer can assist in the identification of probable and confirmed sarcopenia using EWGSOP2 cut off points for low muscle mass in a population of older T2DM patients for further analysis and early treatment. This is notably true in patients possessing high body mass index (BMI) alongside normal ASMI and HGS, potentially reducing false positive sarcopenia screening results.

**Trial registration:**

ClinicalTrials.gov PRS: NCT03560375. Last registration date (last update): 06/06/2018. The trial was a-priori registered before actual recruitment of subjects.

## Introduction

The prevalence of diabetes in the US is steadily increasing, reaching 26.8% (14% in men and 12% in women) among adults aged 65 years or older [1]. The estimated global diabetes prevalence is 19.3%, with North America and the Caribbean possessing the highest regional diabetes prevalence of 27% [2]. Type 2 diabetes mellitus (T2DM) is associated with an accelerated reduction in muscle mass [3], strength [4] function [5], disability [6] and frailty [7], resulting in reduced autonomy and an increased burden on public health care systems [8].

Sarcopenia, characterized by a progressive loss of skeletal muscle mass, strength, and functional abilities [9] is a common complication in older patients with T2DM [10, 11], further increasing their risk for functional decline [12], and physical disability [13]. Compared to the general older population, studies evaluating sarcopenia in older T2DM patients are scarce with prevalence rates varying greatly, ranging from 7 to 29.3% [14]. This disparity is attributed mainly to variations between populations, variations in quantitative evaluation methods, as well as different diagnostic criteria [14, 15].

The European Working Group on Sarcopenia in Older People (EWGSOP2) recently updated its original algorithm for “sarcopenia case-finding”. Muscle strength rather than muscle mass has become the main criteria for diagnosis termed “probable sarcopenia” as muscle strength is the most reliable measure of muscle function [16]. Sarcopenia is further confirmed by the additional detection of a low muscle mass measurement. The EWGSOP advises the use of either the chair rise test, but preferably the use of a handgrip strength test (HGS), due to it its association with functional limitations, and ease of administration in clinical settings.

Older patients with T2DM typically experience a significant loss of lower body strength [17–19] which is associated with a deteriorating health status [20], impaired mobility [21] and loss of autonomy [22]. Isometric knee extension strength testing (KES) using a relatively inexpensive hand-held dynamometer has been found to be valid and reliable in assessing lower body muscle strength with a moderate to high correlation to isokinetic measurements (considered to be the gold standard method) [23–25],especially in older populations [26]. Furthermore, KES has been found to be superior to HGS as an indicator of muscle dysfunction in patients with T2DM [27]. Due to the increased adiposity often accompanying T2DM older patients, it is recommended that knee extension strength relative to bodyweight should be measured as it better relates to low mobility than absolute strength scores [28].

Due to the importance in the early detection and treatment of probable [29] and confirmed sarcopenia, we aim to identify the most appropriate screening strength test by comparing the prevalence of sarcopenia by the use of relative KES to absolute HGS measurements in older patients with T2DM. To this end, we evaluated the association between KES and HGS to appendicular skeletal mass index (ASMI), and the prevalence of low-test scores of sarcopenia parameters and common physical performance screening tests.

## Methods

### Study design

The present investigation is an analysis of baseline measurements of patients enrolled in a randomized clinical trial, which investigated the efficacy of resistance strength training, pharmacotherapy (empagliflozin), and a diet intervention (vegetarian), on the prevention of sarcopenia and/or frailty [30]. The analysis presented here in, is a cross**-**sectional analysis.

### Subjects’ characteristics

Overall, 100 older (≥65 years) male and female T2DM patients, diagnosed in accordance with the American Diabetes Association guidelines were recruited in the original study and their baseline data is presented in the current study (Table 1). The subjects were patients of the various out-patient clinics at the institute of Endocrinology, Metabolism, and Hypertension (IEMH), Tel-Aviv Sourasky Medical Center (TASMC). Eligible patients passed a physician interview and physical examination. A comprehensive survey which included health status, lifestyle as well as physical activity habits were filled in by the patients. The main Inclusion criteria included: Performing ≤2 days a week of any leisure aerobic physical activity, walking independently either with or without an assistance device and HbA1C ≥6.5% to ≤8%. Exclusion criteria included: performing any resistance training within the past six months, the use of anabolic or catabolic steroid agents, severe peripheral neuropathy, end-stage renal failure, history of stroke, myopathy, motor functional disorders and treatment with SGLT-2 inhibitors. The recruitment process as well as the complete eligibility criteria are described in detail elsewhere [30].

**Table 1:** Participants characteristics

Variables	Men (***n*** = 40)	Women (***n*** = 60)	Total (***n*** = 100)
**Anthropometric measurements**			
Age (years)	70.5 ± 4.2	70.4 ± 5.0	70.5 ± 4.6
height (cm)	172 ± 6.1	158 ± 4.5	1.63 ± 0.1
Weight (kg)	94.8 ± 16.4	78.3 ± 14.2	84.7 ± 17.3
WC (cm)	113.5 ± 13.7	106.6 ± 13.6	109.2 ± 14
Body Mass Index (kg/m2)	31.1 ± 5.6	31.4 ± 6.1	31.6 ± 5.9
Lean body mass leg (kg)	9.18 ± 1.35	6.3 ± 0.87	7.35 ± 1.7
Lean body mass arm (kg)	3.64 ± 0.53	2.37 ± 0.46	2.84 ± 0.7
ASMI (kg/m2)	8.55 ± 0.9	6.90 ± 0.9	7.52 ± 1.2
ASM (kg)	25.3 ± 3.36	17.1 ± 4.9	20.36 ± 4.2
Fat mass (%)	36.4 ± 6.7	43.8 ± 5.8	40.8 ± 6.2
**Strength and functional measurements**			
KES (kg)	37.58 ± 8.0	24.0 ± 5.6	29.2 ± 9.4
KES/weight (kg/kg)	0.39 ± 0.07	0.31 ± 0.06	0.34 ± 0.1
HGS (kg)	40.06 ± 6.83	24.97 ± 4.3	31 ± 9.2
HGS/weight (kg/kg)	0.44 ± 0.1	0.33 ± 0.07	0.37 ± 0.1
**Clinical Data**			
Obesity: BMI > 30 (%)	65.0	55.0	59
Obesity: WC > 102 cm men (%) > 88 cm women (%)	87.5	96.6	93
Obesity: > 1 SD than mean reference values of % fat (%) ^1^	77.5	65	70
Diabetes duration (years)	12.3 ± 10.1	13.7 ± 8.8	13.37 ± 9.4
Diabetes> 10 years (%)	58.7	67.8	64
HbA1c (%)	7.63 ± 1.3	7.34 ± 0.9	7.46 ± 1.14
Neuropathy (%)	12.5	17.0	15.2
Nephropathy (%)	15.0	6.8	10.1
Retinopathy (%)	7.7	8.8	8.3
IHD (%)	10.6	5.3	7.4
CHF (%)	5.2	–	2
Hypertension (%)	56.5	54.3	55.2
Statin use (%)	64.1	52.6	57.1
Polypharmacy (> 8 med’s)	25	30	28
Low vitamin D < 25 ng/mL (%)	43.3	43.6	43.4
**Physical performance**			
Gait speed (m/s’)	1.09 ± 0.2	1.08 ± 0.18	1.08 ± 0.19
Timed up & go (s’)	10.6 ± 1.7	10.87 ± 2.16	10.76 ± 2.0
Chair stands 30 s’	11.28 ± 1.8	10.6 ± 2.9	10.99 ± 2.35
2 Min’ walk Test (m’)	172 ± 25.9	158.37 ± 23.65	163.7 ± 25.3

Abbreviations: *ASM* Appendicular Skeletal Mass, *ASMI* Appendicular Skeletal Mass Index, *BMI* Body Mass Index, *CHF* Chronic Heart Failure, *HGS* Hand Grip Strength, *IHD* Ischemic Heart Disease, *KES* Knee Extension Strength, *WC* Waist Circumference

1-According to normative data published [35]

### Muscle quantity and anthropometry

Body composition measurements were obtained through direct segmental multi-frequency bioelectrical impendence analysis (BIA) technique method (*InBody 770 body composition analyzer, InBody Co., Ltd, Seoul, Korea*), by the same technician throughout the study. Measurements included body weight (BW), total and segmental (both legs, trunk, and both arms) skeletal muscle mass, fat mass and % body fat. The In-Body 770 is a valid tool for the assessment of total body and segmental body composition [31]. Height was measured electronically to the nearest 0.1 cm, and body mass index (BMI) was calculated. Waist circumference (WC) was measured using a designated tape measure. ASMI was calculated by adding the sum skeletal masses of both arms and legs divided by height squared using the BIA technique [31]. Low muscle mass was defined according to the EWGSOP2 as ASMI < 7.0 kg/m^2^ in men and < 5.5 kg/m^2^ in women [16].

### Muscle strength assessment

Upper body muscle strength was assessed by a handgrip strength test using a hand-held dynamometer (*Jamar® Sammons Preston Rolyan, Chicago, Illinois, USA*). Subjects were seated in the upright position with the arm along their side, the elbow bent at 90° with the arm supported horizontally by a tester and feet firmly planted on the floor. The width of the dynamometer handle was adjusted to fit the size of the hand (generally using the second smallest grip). One orientation trial was performed for each hand before three trials alternating between arms were performed. The highest of the six measurements was recorded [32]. All trials were separated by a pause of 60 s. Low muscle strength was defined in accordance to the revised EWGSOP2 guidelines as HGS < 27 kg in men and < 16 kg in women [16].

Lower body muscle strength was assessed by an isometric KES test using a hand-held dynamometer (*hydraulic Push-Pull Dynamometer Baseline® evaluation industries*). KES tests using a hand-held dynamometer, have been found to possess both high interrater and intrarater reliability (ICC ≥ 0.95 and ICC = 0.948, respectively) [33]. Participants were asked to sit with their lower legs over the end of a standard examination table, with hips and knees flexed to 90°, and to perform a maximal isometric contraction with the tester’s encouragement. To increase the test’s validity and reliability, the examiner stabilized himself against the subject’s knee extension by positioning one knee on the floor and the other foot against a wall. The dynamometer was placed on the anterior part of the lower leg, just above the talo-tibial joint line, making sure the subject felt no pain, for maximum muscular contraction. A preliminary orientation test was performed on each leg prior to the execution of three 5 s maximal effort measurements, alternating between legs. A minimum pause of 60 s separated between trials and the highest of the six measurements was recorded [23]. Testing procedures, especially hip and knee joint angles were strictly enforced throughout the test. Also, to avoid patient discomfort, a thin foam was placed between the point of the application of the dynamometer and the skin of the patient’s leg. Low muscle strength was defined as the relative KES of KES/BW) ≤ 0.34 kg/bw for men and ≤ 0.24 kg/bw for women (low scores were determined as 1 SD’ below average scores found in a healthy older adult population, *n* = 700) [34].

### Physical performance assessment

#### 4-m gate speed

Patients were asked to walk a 4-m distance at their regular pace. The tester used a hand-held stopwatch to record patients’ completion times. Patients performed one orientation trial before performing two tests and the averaged time was computed. To covert test completion times to gate speed in meters/second (m/s), completion times were divided by 4 (meters). An impaired physical performance was considered a gait speed of 5 s or above corresponding to gait speed of ≤0.8 m/s [16].

#### Timed up and go test (TUG)

Patients were seated in an armchair with a seat height of ~ 44 cm and were timed on their ability to stand up from the chair, walk a 3-m course, turn safely around the cone, walk back, and sit down again (in seconds). The patients performed one preliminary trial before an actual test. A TUG test time of ≥20 s was indicative of impaired physical performance [16].

All muscle strength and physical performance assessments were performed by the same researcher throughout the study. Table 2 summarizes the EWGSOP1–2 guidelines defining cut-offs used to determine sarcopenia.

**Table 2:** EWGSOP1 vs EWGSOP2 sarcopenia cut-off values & operational definitions

EWGSOP1 Cut-off points			EWGSOP2 cut-off points	
Parameter	Men	Women	Men	Women
Gait speed	< 0.8 m/s		< 0.8 m/s	
HGS	< 30 kg	< 20 kg	< 27 kg	< 16 kg
ASMI	< 7.25 kg/m^2^	< 5.67 kg/m^2^	< 7 kg/m^2^	< 5.5 kg/m^2^
**EWGSOP1 definitions**			**EWGSOP2 definitions**	
Pre/Probable Sarcopenia	Low ASMI		Low HGS	
Confirmed Sarcopenia	Low ASMI + Low HGS/ low Gait speed		Low HGS + Low ASMI	
Severe Sarcopenia	Low ASMI + Low HGS + low Gait speed		Low HGS + Low ASMI + Low Gait speed	

Abbreviations: *ASMI* Appendicular Skeletal Mass Index, *EWGSOP1* European Working Group on Sarcopenia in Older People 1, *EWGSOP2* European Working Group on Sarcopenia in Older People 2, *HGS* Hand Grip Strength

### Statistical analysis

Study population characteristics are reported as mean values and standard deviations for continuous variables and percentages for categorical variables**.** Pearson correlations were calculated to evaluate the correlation between muscle mass (ASMI) and muscle strength (KES and HGS), as well as between muscle strength and measures of obesity (BMI, WC, body fat %) (Table 3). A regression analysis using two blocks was conducted to examine which independent variable best predicts ASMI values. In the first block sex and weight were inserted as independent variables to control on the main characteristics of ASMI, using the enter method. The second block contained HGS, KES, vitamin D, HbA1c, performing physical activity, age, years of diabetes, and polypharmacy as independent variables to find which variable significantly influences ASMI after controlling for covariates, as well as the proportion of the predicted variance (R^2^) of ASMI, explained by the independent variables. We used the stepwise method, where at each step, the independent variable with the highest effect on the dependent variable entered the equation (if its *p*-value was < 0.05). The effect of these variables (including sex) on KES and HGS were examined in a multivariate analysis of covariance (MANCOVA).

**Table 3:** Correlations table (between sarcopenia parameters and anthropometric measurements)

Variable	Men	Women
KES / ASMI	*r* = 0.55, *p* < 0.001	*r* = 0.55, *p* < 0.001
HGS / ASMI	*r* = 0.058, *p* = 0.721	*r* = 0.14, *p* = 0.157
KES / BMI	*r* = 0.45, *p* = 0.004	*r* = 0.51, *p* < 0.001
HGS / BMI	*r* = 0.133, *p* = 0.41	*r* = 0.03, *p* = 0.82
KES / body weight	*r* = 0.414, *p* = 0.007	*r* = 0.5, *p* < 0.001
HGS / body weight	*r* = 0.0, *p* = 0.97	*r* = 0.038, *p* = 0.814
KES / body fat %	*r* = 0.22, *p* = 0.19	*r* = 0.37, *p* = 0.004
HGS / body fat %	*r* = −0.26, *p* = 0.1	*r* = − 0.151, *p* = 0.25
KES / WC	*r* = 0.32, *p* = 0.049	*r* = 0.38, *p* = 0.002
HGS / WC	*r* = −0.17, *p* = 0.27	*r* = 0.05, *p* = 0.68
KES / height	*r* = 0.05, *p* = 0.74	*r* = −0.05, *p* = 0.69
HGS / height	*r* = 0.36, *p* = 0.02	*r* = 0.32, *p* = 0.01

Abbreviations: *ASMI* Appendicular Skeletal Mass, *BMI* Body Mass Index, *HGS* Hand Grip Strength, *KES* Knee Extension Strength, *WC* Waist Circumference

## Results

### Participant’s characteristics

#### Clinical characteristics

100 older patients with T2DM (40 men, 60 women) are listed in Table 1. Mean age was 70.5 ± 4.6 years, mean HbA1c was 7.47 ± 1.14 and BMI 31.7 ± 5.95 kg/m^2^. The prevalence of overweight (BMI 25.0–29.99 kg/m^2^) and obesity (BMI > 30 kg/m^2^) in our cohort was 92.5 and 65% in men, and 88 and 55% in women, respectively. The prevalence of central obesity as defined by waist circumference (men≥102 cm and women≥88 cm) was observed in 87.5 and 96.4% of men and women, respectively.

#### Muscle and functional characteristics

Main mean values were (men vs women): ASMI (8.55 ± 0.9 vs 6.90 ± 0.9 kg/m^2^), KES (37.58 ± 8.0 Kg vs 24.0 ± 5.6 Kg) and HGS (40.06 ± 6.83 vs 24.97 ± 4.3 Kg). The prevalence of low skeletal mass defined by EWGSOP2’s ASMI cut-off points for low muscle mass was 7.5% in men and 10% in women. Slow gait speed was found in 7.5% of men (*n* = 3) and 6.7% of women (*n* = 4). Low TUG scores were found in 1.7% of women (*n* = 1) (see Table 1 for EWGSOP1 &2 cut-off points).

### Prevalence of probable and confirmed sarcopenia using HGS and KES cut-off points, low ASMI and low physical abilities (see Table 4 and Fig. 1 for prevalence of sarcopenia)

#### Handgrip

##### According to EWGSOP2

Probable sarcopenia, defined by low HGS was identified in only one patient (woman). Confirmed sarcopenia by low HGS and low muscle mass, was not identified in any of our patients.

**Table 4:** Sarcopenia prevalence, status, and parameters, according to EWGSOP1 and EWGSOP1 definitions

Sarcopenia status	Sarcopenia parameter	EWGSOP1
Probable	Low ASMI	3 men, 7 women (3, 7%)
Confirmed	Low ASMI + Low HGS	1 woman (1%)
Confirmed	Low ASMI + Low KES	2 men, 2 women (2, 2%)
Sarcopenia status	**Sarcopenia parameter**	**EWGSOP2**
Pre sarcopenia	Low HGS	none
Pre sarcopenia	Low KES	13 men, 12 women (13, 12%)

Abbreviations: *ASMI* Appendicular Skeletal Mass Index, *EWGSOP1* European Working Group on Sarcopenia in Older People 1, *EWGSOP2* European Working Group on Sarcopenia in Older People 2, *HGS* Hand Grip Strength, *KES* Knee Extension Strength

##### According to EWGSOP1

Pre-sarcopenia was identified in 10% of patients (three men and seven women). Sarcopenia, defined by low muscle mass and low HGS was identified in one patient (one woman).

#### Knee extension

##### According to EWGSOP2

Probable sarcopenia defined by relative KES was identified in 25% of patients (13 men and 12 women). Confirmed sarcopenic was identified in 3% of patients (two men and one woman). Severe sarcopenia was identified in one patient (man).

#### According to EWGSOP1

Pre-sarcopenia, defined by low muscle mass was identified in 10% of patients (three men and seven women). Sarcopenia defined by low muscle mass and low relative KES was identified in 4% of patients (two men and two women). Severe sarcopenia was identified in one patient (man).

#### Physical performance

The prevalence of patients with low physical performance (gait speed) concurrently possessing low relative KES was found in 66% (*n* = 3) and 75% (*n* = 4) of men and women respectively.

### Correlations of KES and HGS to ASMI

#### Pearson correlation coefficients

KES scores were found to have a moderate correlation to ASMI in men (*r* = 0.551 *p* < 0.001, *n* = 38), (Fig. 2a) and women (*r* = 0.551, *p* < 0.001, *n* = 60), (Fig. 2b). Conversely, HGS was not found to correlate to ASMI in either men (*r* = 0.058, *p* = 0.721, *n* = 40) (Fig. 2c) nor women (*r* = 0.139, *p* = 0.157, *n* = 60), (Fig. 2d). Also, KES but not HGS was found to have a moderate correlation with BMI (*r* = 0.45, *p* = 0.004; *r* = 0.51, *p* < 0.001) and a weak correlation with BW (*r* = 0.41, *p* = 0.007; *r* = 0.5, *p* < 0.001) and WC (*r* = 0.32, *p* = 0.049; *r* = 0.38, *p* < 0.002) in men and women respectively. Body fat % had a weak correlation only to KES and only in women (r = 0.37, p < 0.001). HGS was found to possess a weak correlation to body weight in the total cohort (*r* = 0.41, *p* < 0.001). Height showed a weak correlation to HGS but not to KES in both men (*r* = 0.36, *p* = 0.02) and women (*r* = 0.32, *p* = 0.01), respectively (Table 3). (Interpretation of the Pearson’s correlation coefficients: 0.00 to 0.25 very weak; 0.26 to 0.49 weak; 0.50 to 0.69 moderate; 0.70 to 0.89 strong; 0.90 to 1.00 very strong) [36].

**Fig. 1:**
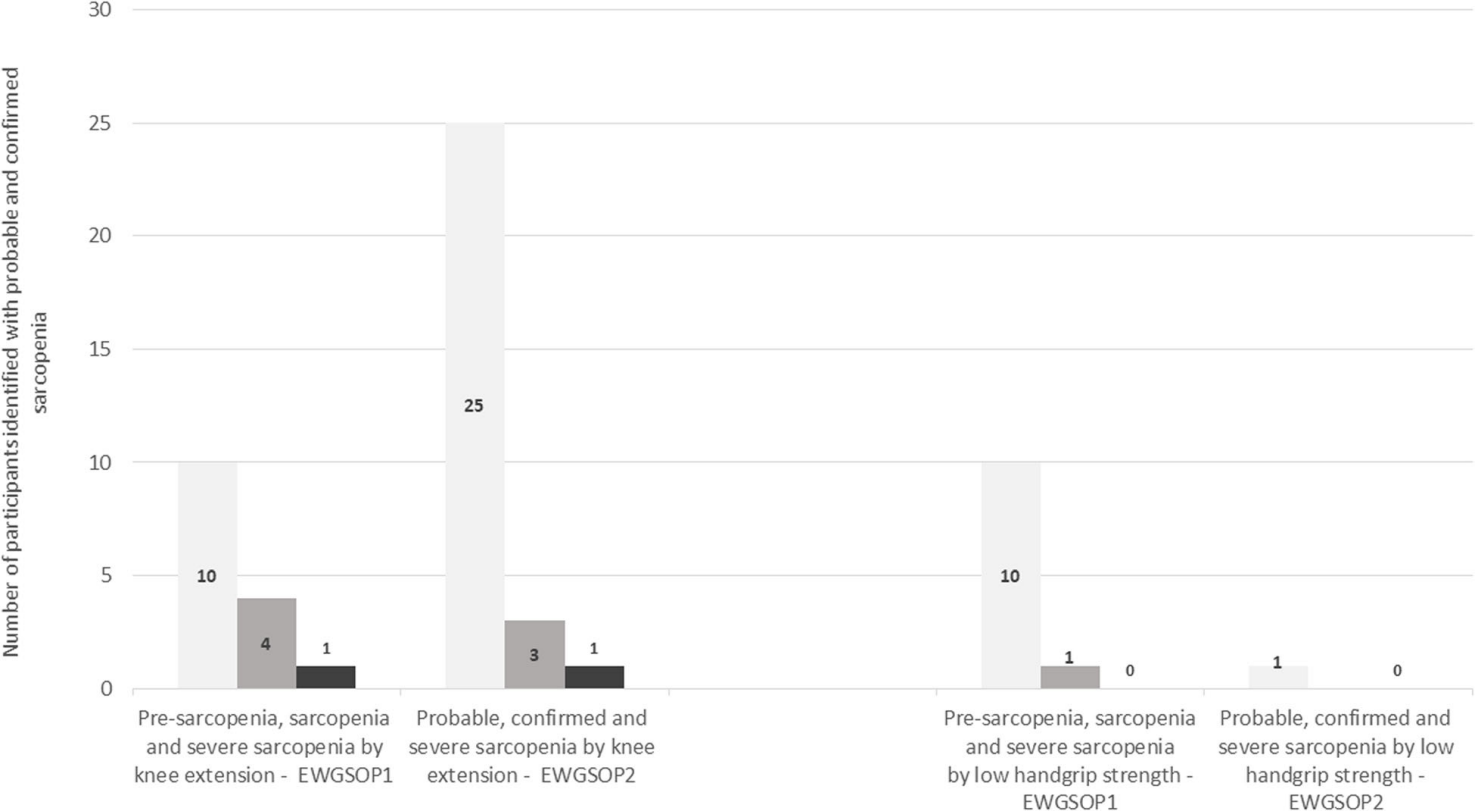
Prevalence of different sarcopenia levels according to EWGSOP1 and EWGSOP2 using low hand grip strength or low knee extension strength

**Fig. 2:**
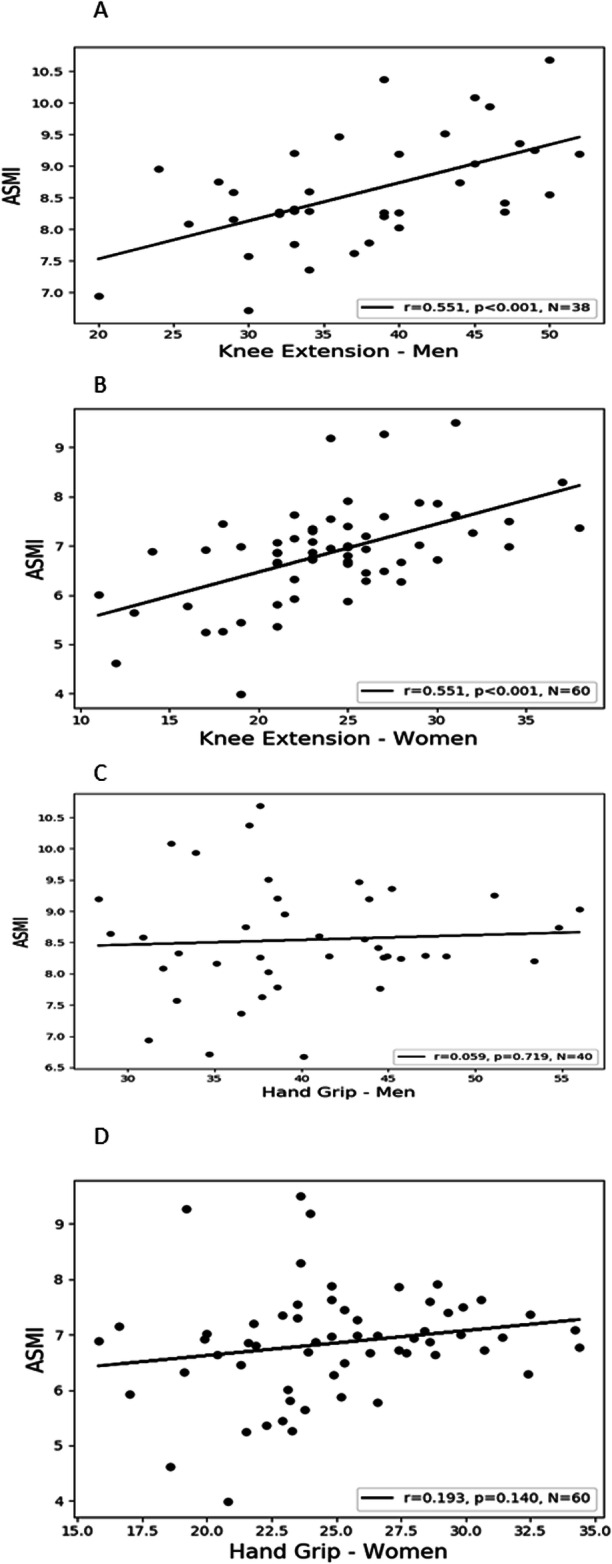
Figure 1A-B: a scatterplot representation of individual knee extension scores and their correlations to ASMI in: **a**) men; **b**) women. Figure 1C-D: a scatterplot representation of individual handgrip scores and their correlations to ASMI in: **a**) men; **b**) women

#### Multivariate analysis

We conducted a linear regression analysis with two blocks to predict the ASMI. In the first block sex and weight were inserted as independent variables to control on the main characteristics of the ASMI, using the enter method. In the second block the stepwise method was used, containing HGS, KES, vitamin D levels, HbA1c, conducting physical activity, age, duration of T2DM and number of medications as independent variables to find which variable significantly influences ASMI after controlling for the covariates (sex and weight). At each step, the independent variable not in the equation possessing the smallest probability of F was entered, if its *p*-value in the model was < 0.05. In the first block, regression analysis revealed that 87.8% of the predicted variance (R^2^) of the ASMI was explained by sex and weight. In the second block, KES was the only variable that entered the model, meaning that after clearing the common variance with sex and weight, HGS (beta in =0.053, *p* = 0.459), vitamin D levels (beta in = − 0.034, *p* = 0.373), HbA1c (beta in = − 0.006, *p* = 0.875), physical activity (beta in = − 0.063, *p* = 0.091), age (beta in =0.042, *p* = 0.281), duration of T2DM (beta in = − 0.008, *p* = 0.858), and polypharmacy (beta in = − 0.011, *p* = 0.770) were not correlated with ASMI. The final model revealed that 88.2% of the predicted variance (*R*^2^) of ASMI could be explained by weight, sex, and KES (*p* < 0.001) (Table 5). The correlation matrix with regression variables can be found in supplementary files.

**Table 5:** Hierarchical multiple regression

Block	Predicting Variable	t	β	F change	R^**2**^ change	F	R^**2**^
**1**	Weight	14.127	.701***			310.351***	0.878
	Sex	−4.272	−.231***				
**2**	Knee extension strenght	2.012	.124*	4.048*	0.005	215.756***	0.882

Parameters in regression model *p < 0.05, **p < 0.01, ***P < 0.001

Note: t- The t statistic is the coefficient divided by its standard error. β is the normalized coefficient, the highest its absolute value, the variable have more influence on the depended variable. The F value in regression is the result of a test where the null hypothesis is that all of the regression coefficients are equal to zero. *R*^2^ - coefficient of determination is a statistical measure of how well the *regression* predictions approximate the real data points

### The effect of independent variables on knee extension strength and handgrip strength

A MANCOVA test was used to examine the association between groups of high and low values of weight (<mean weight), age (< 75), HbA1c (< 7.5), years with T2DM (< 10), Vitamin D levels (< 25), polypharmacy (≥8) and physical activity (yes / no), as independent variables with sex as covariant, to KES and HGS as dependent variables (Table 6). There was a statistically significant difference in KES and HGS based on the weight and years with T2DM. Univariate ANOVAs revealed weight was only associated with KES (F = 6.59, *p* < 0.05), but not with HGS (F = 0.009, NS). Meaning, participants with higher weight had higher KES. Moreover, ANOVAs indicated that both KES and HGS were significantly correlated to duration of T2DM (*p* < 0.01), (*p* < 0.05). Participants with less than 10 years of diabetes had greater KES and HGS.

**Table 6:** Multivariate Analysis of Covariance (MANCOVA) - effects on knee extension and handgrip strength

Variable	Wilks’ λ	f	DF/DF Error	Partial η2
Weight	0.915	3.562*	2,77	0.085
Age	0.945	2.221	2,77	0.055
T2DM Years	0.887	4.925*	2,77	0.113
HbA1c	0.988	0.462	2,77	0.012
Vitamin D	0.987	0.512	2,77	0.013
# of drugs	0.998	0.077	2,77	0.002
Physical Act	0.987	0.492	2,77	0.013

*p < 0.05

Note: Wilks’ λ represents the ratio between the error variance (or covariance) and the effect variance (or covariance), F is the statistics, DF – degree of freedom, Partial η2 - effect size, represents the proportion of the variance in the dependent variable that can be explained by the variance in the groups of a categorical independent variable

## Discussion

The main finding of our study was that cut-off points for low KES identified considerably more patients with probable and confirmed sarcopenia compared to HGS testing using the EWGSOP2 cut-off points for low HGS. The low prevalence of sarcopenia using HGS cut-off points in older adults with T2DM found in our study (3%) was also observed by Villani et al. [37] identifying 2.3% of his T2DM older patients with confirmed sarcopenia (age eligibility ≥50 years) and Freitas’s et al. [38] identifying a somewhat higher prevalence rate of confirmed sarcopenia (7%) and mostly women (88%), potentially due to a higher ASMI cut-off point for women (6.0 kg/m2). This observation is perplexing since T2DM older patients are known to possess markedly reduced muscle strength [5], and in particular low handgrip strength [39], with an increased risk for sarcopenia [40, 41].

Despite the EWGSOP2 algorithm’s potential to reduce health costs by reducing the number of DXA measurements to identify sarcopenic patients [42], it has been often scrutinized by investigators, observing a markedly lower prevalence of probable and confirmed sarcopenia compared to the earlier EWGSOP1 guidelines. This lower prevalence of sarcopenia is mainly due to the reduced cut-off points for both HGS (3 k“g and a 4 k”g reduction in HGS for men and women respectively), and ASMI (a 0.25 kg/m^2^ and 0.17 kg/m^2^ reductions in men and women respectively [42–45]. Indeed, in our cohort of older T2DM patients, a higher prevalence of mostly pre-sarcopenia (10%) and confirmed sarcopenia (4%) was identified using the EWGSOP1 cut-off points for low HGS.

The EWGSOP2 recommends the use of one or two strength tests as their primary criterion for the identification of sarcopenia, an upper body test (HGS) and a lower body test (five-repetition chair stands) [16]. We believe that the addition of a lower body strength test to the HGS test is imperative since the HGS is a poor predictor of both total body strength [20], and functional performance [46, 47], while yielding dissimilar sarcopenia rates compared to lower body testing (chair stands), in a community-dwelling group of middle aged and older adults [48]. The KES test can be recommended as an alternative lower body strength test for the chair rise test, as it involves less complex weight-bearing body movements [49], is better suited for diabetics with peripheral neuropathy [50], as well as obese older adults with moderate to advanced osteoarthritis [51, 52].

Our study was not exclusive in observing that the identification of probable sarcopenia is dependent on the strength tests performed. Wearing et al. has shown that by choosing a proper strength screening test 27% of patients would benefit from the continuation of the screening process, possibly preventing further strength reductions through an early initiation of suitable interventions [29]. The higher prevalence of probable sarcopenia in measures other than HGS was found in other studies using the new EWGSOP2 algorithm. Higher rates of probable sarcopenia using the chair rise test were found by Kim et (13% in women) [53], as well as Johansson et al. (4.4% vs 1.3%) [48], noticing that subjects identified with probable sarcopenia by chair raise, were heavier and more obese than subjects identified by HGS, probably due to the greater influence of relative leg muscle strength. Dodd et al. observed that chair rise detected double the prevalence rates of probable sarcopenia in comparison to the HGS (15% vs 7%), stating the need to perform both tests to better assess probable sarcopenia [54].

The correlation found between KES and ASMI along with other body size measures (BMI, body weight, WC) is important as it can be associated with an “obesity paradox”. The obesity paradox is generally referring to the protective effect obesity imparts on decreased mortality in older adults [55]. That said, the term obesity paradox has also been related to the added muscle mass [11, 56] and muscle strength [57], Caused by the anabolic effect that occurs through continuously carrying the added body mass associated with obesity. This phenomenon is especially expressed in the lower body [58], through an increase in absolute KES [59], underscoring the need to normalize KES to body size using relative strength cut-off points. Relative muscle strength (muscle strength divided by bodyweight) better identifies individuals with reduced muscle strength through either HGS [60, 61] or KES testing [28, 62], thus possibly reducing false negative sarcopenia assessments.

The high prevalence of obesity in our cohort, can possibly explain the low prevalence of confirmed sarcopenia through low ASMI scores that coincide with the higher BMI values generally associated with higher fat free mass [63].

Overall, the prevalence of common health complications exhibited by the subjects in our study was somewhat lower than values generally seen in older T2DM patients [64]. Our finding that muscle strength (both HGS and KES) was significantly correlated to duration of T2DM is in line with chen et al. [62], while unlike Izzo et al. [14], stating that diabetes duration does not increase the prevalence of sarcopenia. The finding that most of the patients possessing low physical performance scores, concurrently possess low relative KES compared to those with impaired HGS, further affirms the added value in using KES as a sarcopenia screening tool.

Our study has several limitations. Its main limitation is the relatively small sample of T2DM patients making our findings difficult to generalize to older adults with T2DM. Additionally, the majority of our patients were younger than 75 years of age with only a small number of patients being older than 75, thus reducing the potential to truly investigate the age factor on outcome measures.

## Conclusion

In a cohort of 100 mostly obese T2DM older patients, relative KES cut-off points using a simple hand-held dynamometer can assist in the identification of mostly probable sarcopenia and confirmed sarcopenia cases using EWGSOP2 cut off points for low muscle mass. Using the EWGSOP2 cut off points for low muscle strength by HGS mostly failed to identify probable and confirmed sarcopenia, possibly due to high prevalence of normal absolute handgrip and ASMI values associated with subjects possessing high BMI and body weight.

It must be stated that our study is the first to assess the prevalence of probable and confirmed sarcopenia in older T2DM patients by relative KES cut off points. Due to the importance of the initial screening strength test for further analysis and early treatment of sarcopenia and to reduce false negative sarcopenia, it would be prudent to add a lower body strength test such as the relative KES while screening older T2DM patients for sarcopenia while using the EWGSOP2 guidelines.

## Data Availability

The datasets generated and/or analyzed during the current study are not publicly available due to institutional restrictions but are available from the corresponding author on reasonable request.

## Abbreviations

ANOVA: one-way analysis of variance
ASMI: appendicular skeletal mass index
BIA: bioelectrical impendence analysis
BMI: body mass index
EWGSOP 1/2: The European Working Group on Sarcopenia in Older People
HGS: handgrip strength
KES: knee extension strength
MANCOVA: multivariate analysis of covariance
T2DM: Type 2 Diabetes Mellitus
TUG: timed up and go test
WC: Waist circumference
